# A purinergic P2Y6 receptor agonist prodrug modulates airway inflammation, remodeling, and hyperreactivity in a mouse model of asthma

**DOI:** 10.2147/JAA.S151849

**Published:** 2018-08-01

**Authors:** Anne Chetty, Azeem Sharda, Rod Warburton, Ellen O Weinberg, Jinghui Dong, Min Fang, G Gary Sahagian, Tiangmeng Chen, Chang Xue, John J Castellot, Philip G Haydon, Heber C Nielsen

**Affiliations:** 1 Department of Pediatrics, Tufts Medical Center, Boston, MA, USA, Heber.nielsen@tufts.edu; 2 Department of Medicine, Tufts Medical Center, Boston, MA, USA; 3 Department of Integrative Physiology and Pathobiology, Tufts University School of Medicine, Boston, MA, USA; 4 Department of Neuroscience, Tufts University School of Medicine, Boston, MA, USA; 5 Department of Developmental, Molecular and Chemical Biology, Tufts University School of Medicine, Boston, MA, USA; 6 Graduate Program in Cell, Molecular and Developmental Biology, Tufts University School of Medicine, Boston, MA, USA, Heber.nielsen@tufts.edu

**Keywords:** airway smooth muscle, pulmonary function, inflammation, cytokines, P2Y6 receptors, GC021109

## Abstract

**Background:**

Purinergic receptors control cell proliferation, apoptosis, migration, inflammation, and cytokine secretion. Increased expression of specific purinergic receptors is reported in asthma. The role of purinergic P2Y6 receptors (P2Y6R) in asthma is controversial.

**Hypothesis:**

P2Y6R activation in asthma improves pulmonary function and reduces inflammation and smooth muscle amount.

**Methods:**

Female mice (C57/BL6, age 30 days) were randomly assigned to receive intranasal house dust mite (HDM) antigen (40 or 80 µg) or saline, 5 days/week, for 6 weeks. Randomly selected subgroups received intraperitoneal P2Y6R agonist prodrug (GC021109; 10 or 100 µg/kg weight/dose) simultaneously with HDM. After 6 weeks, lung function was measured. Lung lavage fluid (LLF) was used to measure total cell count, total protein, and cytokines. Immunohistochemistry for alpha smooth muscle actin (α-SMA) was done. Airway wall thickness was measured on micro-computed tomography (micro-CT) images.

**Results:**

Pulmonary function testing revealed a HDM dose-dependent airway hyperresponsiveness. Airway resistance was increased 2-fold while compliance was decreased by 50% at the higher HDM dose (*P*<0.05). GC021109 prevented these changes. HDM-exposed mice had elevated inflammatory cell and total protein levels in LLF which were prevented by GC021109 (*P*<0.05). HDM mice also had elevated LLF levels of interleukin (IL)-4, IL-5, IL-12, granulocyte colony stimulating factor, chemokine (C-X-C) motif ligand 1, and leukemia inhibitory factor that were reduced by GC021109 with a dose-dependent pattern. HDM mice had increased peribronchial and perivascular inflammatory cell infiltration and increased α-SMA; these changes were absent with GC021109. Airway wall thickness measured on micro-CT images was increased after HDM exposure and significantly reduced by GC021109 treatment.

**Conclusion:**

The P2Y6R prodrug GC021109 inhibited allergen-induced changes in pulmonary function, inflammatory responses, and airway and vascular smooth muscle mass. P2Y6R activation may be an effective therapeutic maintenance strategy in asthma.

## Plain language summary

Improvements in severe allergic asthma may be achieved by treatments that reverse the underlying changes in the lung. This study shows that a prodrug GC021109 that activates purinergic P2Y6 receptors (P2Y6R) effectively reduces the development of asthma pathology in an established mouse model of asthma. Lung function testing, several markers of airway inflammation, and hypertrophy of airway and lung vascular smooth muscle were all significantly improved when GC021109 was given during the development of asthma. P2Y6R activation may be a useful therapy in chronic asthma management.

## Introduction

Asthma is a chronic lung disease characterized by airway hyperresponsiveness, inflammation with increased mucus secretion, and structural changes in the lungs. Patients with asthma have increased levels of proinflammatory cytokines in bronchoalveoar lavage fluid.^1^ Altered immune responses to common allergens like pollens and house dust mites (HDM) induce production of interleukins (ILs) including IL-4, IL-5, IL-6, and IL-13. Upon recognition of antigen and activation naïve T-cells differentiate into type 2 T helper (Th2) cells, a process initiated by IL-4. Cytokines released by Th2 cells promote the release of mucus and chemokines from airway epithelial cells, setting up a perpetuation of airway inflammation by further promoting IL-12, IL-5, IL-6, and granulocyte-macrophage colony stimulating factor (GM-CSF). These events cause the accumulation of eosinophils, macrophages, and neutrophils which induces structural and functional changes in airways. These changes include epithelial cell shedding, basement membrane thickening, and airway smooth muscle (ASM) cell proliferation and hypertrophy. The hypertrophied ASM has an exaggerated contractile response to proinflammatory cytokines and other contractile stimulants, causing acute narrowing of the airways that restricts bronchial and bronchiolar air flow. In addition, bronchial vascular remodeling occurs with increases in the size and number of blood vessels leading to vascular hyperemia, and increased vascular smooth muscle.^2^,^3^ Clinical therapy for asthma is focused on reducing the chronic inflammation and reversing acute bronchoconstriction.

Purinergic receptors are a superfamily of cell membrane receptors that are activated by purines. The P2Y6 purinergic receptor belongs to the family of P2 receptors which consist of P2X ligand-gated ion channels and P2Y G-protein coupled receptors. The P2 receptor family has several receptor subtypes with different and overlapping pharmacological activities. The P2Y6 receptor (P2Y6R) is responsive to uridine diphosphate (UDP), uridine triphosphate, and adenosine diphosphate but not to adenosine triphosphate. Purinergic receptors are present in all tissues and regulate cell proliferation and migration, induce changes in vascular tone to affect blood flow, and induce cytokine secretion. The specific roles of P2Y receptor subtypes, particularly those activated by pyrimidines, are not well established. Most P2Y6Rs lack potent and selective agonists and antagonists. P2Y6Rs can be activated by nucleotides released during inflammation, leading to further inflammatory cytokine release.^4^

The involvement of P2Y receptors in inflammation during asthma has been studied in animal models and in humans, with varying results.^5^–^8^ P2Y6R activation was reported to actively promote inflammation in asthma.^5^ However, P2Y6Rs may also exert anti-inflammatory activity. For example, P2Y6R activation protects against allergen-induced lung inflammation by inhibiting T-cell activation.^9^ Overall, the function during asthma of P2Y receptors, particularly of P2Y6R, is incompletely understood. To learn more about the functional significance of P2Y6R activity in asthma, we used chronic administration of a small molecule P2Y6R agonist prodrug (GC021109) in a juvenile mouse asthma model.

In independent studies, small molecule agonists selective for P2Y6R have been developed in a medicinal chemistry campaign. These molecules exhibit high selectivity for P2Y6R over closely related P2Y2 and P2Y4Rs. In addition, prodrug forms of these agonists have been developed which exhibit excellent drug-like capabilities including bioavailability. In addition to exerting positive outcomes in preclinical studies of Alzheimer’s disease, the lead candidate GC021109 has been shown to exert anti-inflammatory effects. Thus, this compound, which has been used in clinical trials where it has been shown to be safe and well tolerated, was chosen to determine whether it prevents allergen-induced asthma.

## Methods

### Animals

One-month-old female mice (C57/BL6) were purchased from Taconic Farms, NY, USA and housed at Tufts Medical School laboratory animal facility. This facility conforms strictly to the current National Institutes of Health guidelines for animal care. The animal use protocol was approved by the Tufts University and Tufts Medical Center Institutional Animal Care and Use Committee.

### HDM sensitization: chronic HDM model

HDM antigen is one of the most common aeroallergens causing clinical allergy and asthma. Mice given chronic nasal administration of HDM develop an asthmatic phenotype that reproduces many physiologic components of human asthma.^10^,^11^ We used this well-established model of allergic asthma by treating mice with intranasal administration of 40 or 80 µg of *Dermatophagoides pteronyssinus* (Greer Laboratories, Lenoir, NC, USA) dissolved in 25 µL of saline or saline alone (vehicle) by pipetting drops onto the nostrils. HDM administration was begun at 1 month of age and continued 5 days/week for 6 weeks.

### GC021109 administration

GC021109 was supplied by GliaCure, Inc (Boston, MA, USA). It is a nucleoside that does not activate the P2Y6R nor over 70 channels, transporters, or receptors assayed in an off-target screen. However, when administered to cells in vitro, or when delivered in vivo, the nucleoside is phosphory-lated to a nucleotide (GC011002) that selectively activates P2Y6R (EC50 20 nM). Like the endogenous agonist UDP, once the nucleotide agonist GC011002 is released from cells it is rapidly hydrolyzed by endogenous ectonucleotidases back to the original nucleoside. GC011002 requires the expression of the P2Y6R for activity. In an off-target screen of >70 receptors, channels and transporters, GC021109 was found to be selective for P2Y6R (Philip G Haydon, personal communication, January, 2018).

Randomly selected mice from the nasal saline and the nasal HDM groups received intraperitoneal injections of GC021109 at either 10 or 100 µg/kg weight/dose on the same days that HDM or saline treatments were given.

### Lung mechanics

One day after the end of the 6-week treatment period, groups of 5–6 mice per condition were deeply sedated with ketamine (100 µg/kg weight/dose) and xylazine (10 µg/kg weight/dose) for pulmonary function studies. The trachea was cannulated, the cannula connected to a computer-controlled small animal ventilator (flexiVent; SCIREQ, Montreal, QC, Canada), and ventilation delivered at a frequency of 150 breaths/minute and tidal volume of 5 mL/kg.^11^ Pulmonary function parameters in response to increasing methacholine (MCh) doses (25–200 mg/mL) were measured. The frequency-independent airway resistance (cm of H_2_O/mL/s), elastance (cm of H_2_O/mL), and compliance (C_L_/mL/cm of H_2_O) were calculated using the SCIREQ software as described.^12^,^13^

### Inflammatory responses

Lung lavage was performed only in mice that did not have pulmonary function testing. Groups of 5–6 mice within each treatment were tracheostomized. PBS (0.3 mL) was instilled into the cannulated trachea 3 times, gently withdrawing after each instillation. The 3 returned aliquots were pooled. Total cell number in the lung lavage fluid (LLF) was counted by a hemocytometer. Protein concentration was measured by bicinchoninic acid assay (Thermo Fisher Scientific, Waltham, MA, USA). Cytokine concentration in the LLF was measured in duplicate using a Cytokine Multiplex 32-Plex Discovery Assay (Mouse Cytokine Array/Chemokine Array; Eve Technologies, Calgary, AB, Canada). Duplicates were averaged for the final value.

After lung lavage, the lungs were inflation fixed at 20 cm H_2_O inflation pressure in 4% paraformaldehyde and embedded in paraffin. The fixed tissue was used to prepare tissue sections for histochemical and immunocytochemical analysis. For cytochemical staining, 5-µm sections were deparaffinized in xylene, rehydrated, and washed in PBS. The sections were stained with hematoxylin/eosin and inflammatory cell infiltration viewed by light microscopy.

### Alpha smooth muscle actin (α-SMA)

Sections of fixed lung (5 µm) were deparaffinized in xylene, rehydrated, and washed in PBS. Endogenous peroxidase activity was quenched by incubating sections in 3% hydrogen peroxide (in PBS) for 10 minutes. Sections were blocked with 1.5% goat serum in PBS for 1 hour. α-SMA expression was identified by incubating with alpha α-SMA antibody (Abcam, Cambridge, UK) (1:200) overnight at 4°C followed by biotinylated anti-rabbit IgG (1:400) for 2 hours at room temperature, then with AB enzyme reagent and counter-stained with hematoxylin. Slides were reviewed from at least 3 different animals for each condition. Area measurement of α-SMA staining surrounding intermediate-sized airways and blood vessels was carried out in slides from at least 3 animals per condition using ImageJ (National Institutes of Health, Bethesda, MD, USA). The results were expressed as the ratio to the internal diameter of the respective airway or blood vessel in arbitrary units.

### Micro-computed tomography (micro-CT) imaging and analysis

Micro-CT imaging of the thorax was performed on live animals using a Bruker SkyScan micro-CT system (Bruker SkyScan, Kontach, Belgium) as described by Lederlin et al with minor modifications.^14^ Twenty-four hours after the end of the 6-week treatment period, groups of 3–4 mice per condition were deeply sedated with ketamine (100 µg/kg weight/dose) and xylazine (10 µg/kg weight/dose). Mice were intubated and ventilated (flexiVent) with respiratory gating. The output signal of the chest wall movement was used to allow data acquisition to be triggered at a peak inspiratory pressure hold (IPH) maneuver with the ramp duration of the IPH set to 0.5 seconds and the hold time set to 1 second with the trigger lasting for 0.7 seconds. Images were obtained without any contrast agent at 25–80 mGy per scan. The complete data acquisition required 20 minutes per animal. Anatomical landmarks were used to identify the third-generation bronchi on coronal images, and the corresponding transverse axis images were then used to image the third-generation bronchus in cross section. Airway wall thickness, consisting of the smooth muscle and airway epithelial layers, was measured in the third-generation bronchi in 3 locations per mouse.^15^ Only images in which the bronchus was circular as opposed to ovoid were used for measurements, to avoid image angle distortion. Circularity was established by consistency of the thickness measurement at different positions around the airway.

### Statistical analysis

Results were expressed as mean ± standard error of means of N=4–6 mice (in one experimental set, a mouse died while initiating pulmonary function testing). Statistical analyses were performed by analysis of variance (ANOVA); differences between specific groups were then tested using either reduced model ANOVA or post hoc multiple comparison testing (Dunnett or Bonferroni methods) using GraphPad InStat software (GraphPad Software Inc, La Jolla, CA, USA).

## Results

### Lung mechanics

#### Airway resistance

**Figure 1:**
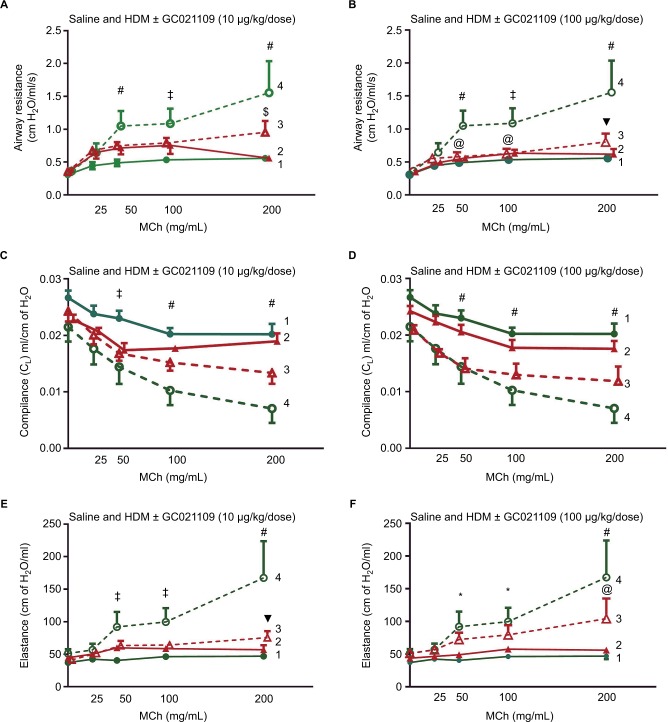
Pulmonary function mechanics are improved in GC021109-treated mice. **Notes:** Acute MCh-induced pulmonary function response curves in 10-week-old mice after 6 weeks of treatment with saline, GC021109, HDM (80 µg), or HDM + GC021109 at either 10 or 100 µg/kg weight/dose. Pulmonary resistance in the **(A)** GC021109 (10 µg/kg weight/dose) treatment group and **(B)** GC021109 (100 µg/kg weight/dose) treatment group. Pulmonary compliance in the **(C)** GC021109 (10 µg/kg weight/dose) treatment group and **(D)** GC021109 (100 µg/kg weight/dose) treatment group. Pulmonary elastance in the **(E)** GC021109 (10 µg/kg weight/dose) treatment group and **(F)** GC021109 (100 µg/kg weight/dose) treatment group. The mice treated with HDM alone exhibited significant changes in pulmonary resistance, compliance, and elastance curves in a pattern consistent with asthma. The combination of HDM and GC021109 reversed these changes. The effect appeared stronger with the 100 µg/kg weight/dose GC021109 dose. GC021109 given with saline was not different from saline alone. Treatment groups: 1 – saline, 2 – saline + GC021109, 3 – HDM + GC021109, 4 – HDM. Values are mean ± SEM of N=4–5 mice per group. *P<0.05, saline control compared to HDM; ^@^*P*<0.05, HDM + GC021109 compared to HDM; ^$^*P*<0.01, HDM + GC021109 compared to HDM; ^‡^*P*<0.01, saline control compared to HDM; ^▼^*P*<0.005, HDM + GC021109 compared to HDM; ^#^*P*<0.005, saline control compared to HDM. **Abbreviations:** HDM, house dust mite; MCh, methacholine; SEM, standard error of means.

Mice sensitized to HDM developed significant reactive airway disease, seen as a progressively increasing airway resistance in response to increasing MCh doses.^11^,^13^,^16^ As expected, mice which received no HDM exposure exhibited a modest dose-dependent increase in airway resistance in response to MCh. Moreover, exposure to GC021109 alone (no HDM sensitization) did not significantly affect the MCh response. However, GC021109 given at a low (10 µg/kg weight/dose) or a high (100 µg/kg weight/dose) dose attenuated HDM-induced changes in airway resistance. MCh-induced airway resistance was significantly blunted by GC021109 (Figure 1A and B).

#### Airway compliance

Mice exposed to saline alone or GC021109 alone exhibited no significant changes in lung compliance. Reactive airway disease is characterized by reduced lung compliance in response to MCh. In agreement with this, lung compliance in response to MCh was significantly reduced in the mice exposed to HDM alone in the characteristic MCh dose-dependent pattern. GC021109 prevented these HDM-associated changes in lung compliance (Figure 1C and D).

#### Airway elastance

Asthmatic airways respond to MCh with an increase in elastance. In these studies, the HDM-exposed mice demonstrated a typical MCh dose–response of increased elastance, while untreated mice and mice that received GC021109 alone demonstrated no significant response to MCh. The mice that were treated with both HDM and the P2Y6R agonist demonstrated a markedly blunted response to MCh (Figure 1E and F).

### Lung immune response

#### Inflammatory cell recruitment

**Figure 2:**
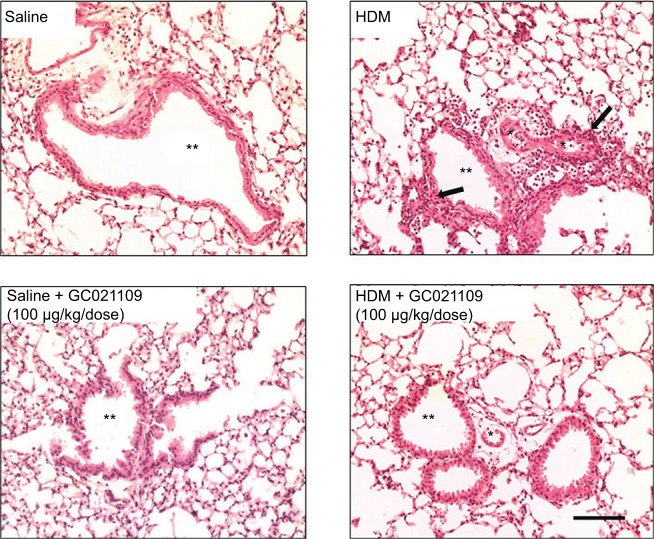
GC021109 prevents HDM-induced peribronchiolar and perivascular inflammatory cell infiltration. **Notes:** Representative photomicrographs of hematoxylin/eosin-stained lung (5-µm sections) from mice treated 6 weeks with either saline, HDM, saline + GC021109 (100 µg/kg weight/dose), or HDM + GC021109 (100 µg/kg weight/dose). Arrows point to inflammatory cell infiltration in the peribronchiolar and perivascular space. Inflammatory cells were prominently increased by HDM treatment. This response was absent in mice treated concomitantly with HDM and GC021109. *Vascular lumen, **bronchiolar lumen. Bar represents 50 µm. **Abbreviation:** HDM, house dust mite.

Lung inflammation was examined in several ways. Hematoxylin and eosin-stained slides were examined for the presence of peribronchiolar and perivascular inflammatory cell infiltrates. Lungs from the saline-treated and from the GC021109-treated mice showed rare peribronchiolar and perivascular macrophages and neutrophils. Lungs from HDM-treated mice had extensive peribronchiolar and perivascular inflammatory cell infiltrates. Addition of GC021109 with HDM significantly modified the cellular inflammatory response, markedly decreasing the presence of inflammatory cell infiltrates around both airways and blood vessels (Figure 2).

**Figure 3:**
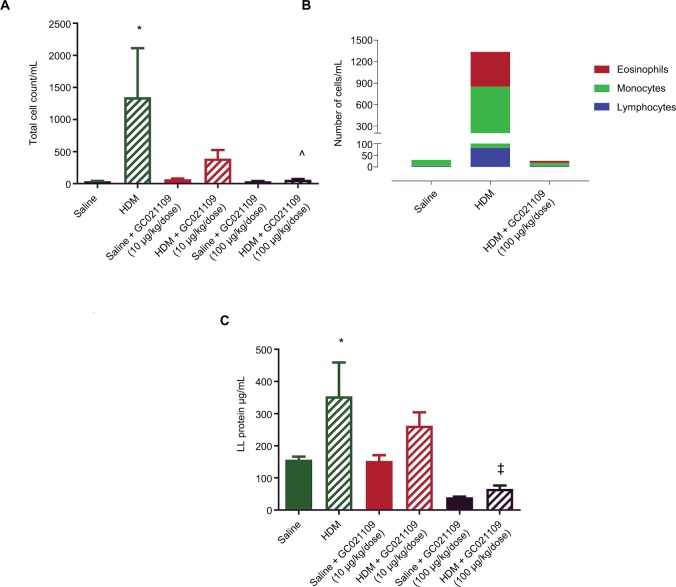
Increases in lung lavage content of inflammatory cells and protein after HDM exposure are normalized by the addition of GC021109. **Notes: (A)** Total inflammatory cell numbers in lung lavage fluid from mice after exposure to saline (solid green), HDM (green striped), GC021109 (10 µg/kg weight/dose – solid red, 100 µg/kg weight/dose – solid black), and HDM + GC021109 (10 µg/kg weight/dose – striped red, 100 µg/kg weight/dose – striped black). Counts were performed using a hemocytometer. **(B)** Cumulative differential cell counts in lung lavage from saline-treated, HDM-treated, and HDM + GC021109 (100 µg/kg weight/dose)-treated mice. **(C)** Protein concentration in lung lavage fluid. Conditions were the same as in **(A)**. Data represent mean ± SEM; N=5 mice per group. *P<0.01, compared to control; ^^^*P*<0.01, compared to HDM; ^‡^*P*<0.001, compared to HDM. **Abbreviations:** HDM, house dust mite; LL, lung lavage; SEM, standard error of means.

In addition to qualitative observations of tissue infiltration by inflammatory cells, we used quantitative approaches to measure the degree of inflammatory cell recruitment by evaluating numbers and types of cells in LLF. The lungs underwent tracheal/bronchial lavage with 0.30 mL of saline, repeated 3 times. Overall, at least 75% of the total saline volume instilled for lavage was recovered; this did not differ among the treatment groups. Lavage fluid from normal lungs typically contains a minimal protein concentration and a minimal number of cells. In agreement with this, the lavage fluid from the mice who received saline or GC021109 alone had very low protein concentrations and cell counts. HDM-exposed mice had a significantly higher protein concentration and recruited significantly more inflammatory cells into the airways (Figure 3A and B). Most cells making up this increase were lymphocytes; a marked increase in eosinophils was also present (Figures 3B and S1). This increase in inflammatory cell content was markedly reduced in animals which received HDM + GC021109 (at both 10 and 100 μg/kg weight/dose). Both the lymphocyte and eosinophil counts in the LLF from mice exposed to HDM + GC021109 were markedly affected, although the differential cell count did not completely return to control levels. The increase in LLF protein content present in the HDM-exposed mice was also reduced to near normal control levels with GC021109 treatment (both 10 and 100 μg/kg weight/dose; Figure 3C).

#### LLF cytokines

**Figure 4:**
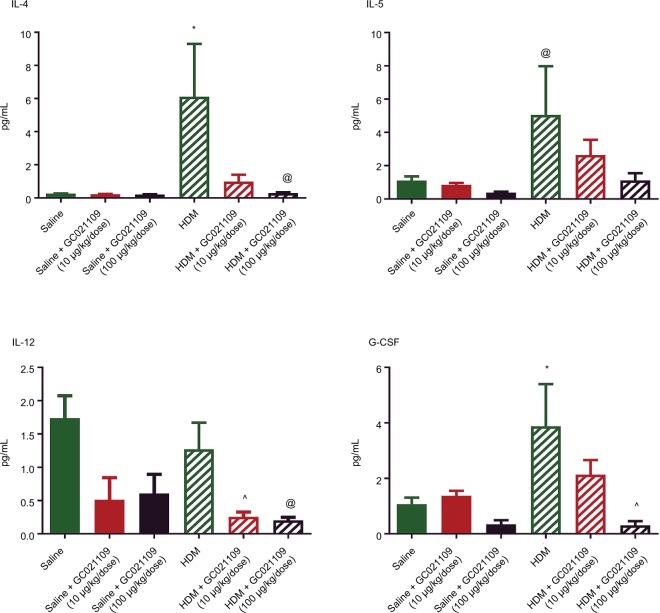
Asthma-related cytokines in lung lavage fluid are increased after HDM exposure but reduced by the addition of GC021109 in a dose-dependent pattern. **Notes:** Cytokines were measured in lung lavage fluid by multiplex cytokine immunoassay. The figure shows the levels of IL-4, IL-5, G-CSF, and IL-12 in lavage fluid from mice treated with saline, GC021109, HDM, and HDM + GC021109 (10 and 100 µg/kg weight/dose). GC021109 alone did not alter the lung lavage cytokine levels as compared to saline, although IL-12 levels in the saline group appeared elevated. IL-4, IL-5, and G-CSF were strongly increased in HDM-treated mice, while IL-12 remained high. Concomitant treatment with HDM and GC021109 (10 or 100 µg/kg weight/dose) produced a dose-dependent reduction in all 4 cytokines. Data represent mean ± SEM; N=5 mice per group. *P<0.05, compared to control; ^^^*P*<0.05, compared to HDM; ^@^*P*<0.10, compared to HDM. **Abbreviations:** HDM, house dust mite; SEM, standard error of means; IL, interleukin; G-CSF, granulocyte colony stimulating factor.

We used a multiplex system to measure a panel of cytokines in the LLF of saline-treated, HDM-treated, and HDM + GC021109-treated mice. Compared to values from the saline-treated mice, HDM treatment significantly increased several cytokines, particularly IL-4, IL-5, IL-12, and granulocyte colony stimulating factor (G-CSF; Figure 4). The concentration of IL-12 (p40) was elevated in saline-treated controls above the level in HDM-exposed mice. This value appears to be an outlier compared with the rest of the treatment groups and their pattern of responses and was not considered in the subsequent analysis. IL-12 was significantly lowered by the combination of HDM and GC021109 when compared to HDM alone. Overall, the levels of these 4 cytokines in HDM-exposed mice were significantly reduced by the addition of GC021109 treatment, usually exhibiting a dose-dependent response pattern. Similar changes with added GC021109 were also observed for IL-6, IL-17, chemokine (C-X-C) motif ligand 1 (CXCL1), and leukemia inhibitory factor, albeit with a less-evident dose–response relationship (Figure S2).

### Airway remodeling

**Figure 5:**
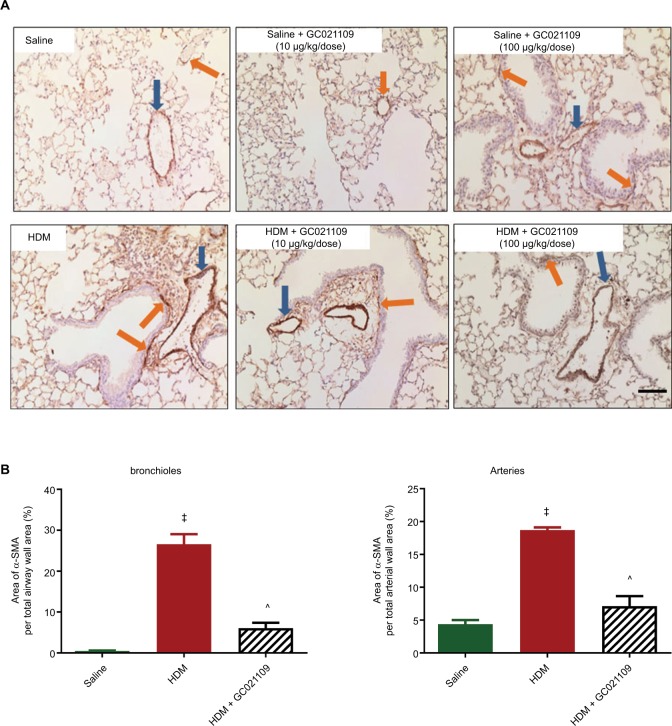
GC021109 reduces HDM-induced increases in peribronchial and perivascular α-SMA. **Notes: (A)** Representative photomicrographs of α-SMA immunostaining in lungs from mice exposed to saline, GC021109 (10 and 100 µg/kg weight/dose), HDM, and HDM + GC021109 (10 and 100 µg/kg weight/dose). Arrows point to α-SMA immunostaining around bronchioles (orange) and blood vessels (blue). Increased α-SMA expression was observed in HDM-exposed lungs compared to controls but not in HDM + GC021109 (both doses). Bar =50 µm. **(B)** Quantification of the relative area of α-SMA immunopositivity around bronchioles and arteries, expressed as a percentage of the total area of the peri-luminal muscle layer. Area measurements were made using ImageJ (National Institutes of Health). HDM treatment significantly increased, and HDM + GC021109 (100 µg/kg weight/dose) prevented increase in α-SMA expression. Data represent mean ± SEM; N=4 lungs (3 measurements each). ^‡^*P*<0.0001, compared to control; ^^^*P*<0.005, compared to HDM. **Abbreviations:** HDM, house dust mite; SEM, standard error of means; α-SMA, alpha smooth muscle actin.

A major anatomical feature of asthma is airway remodeling, characterized by increased smooth muscle cell layers surrounding bronchi and bronchioles. We assessed ASM using immunohistochemistry for α-SMA expression. Control lungs showed minimal staining for α-SMA around small blood vessels and airways. In contrast, lungs from the HDM group of mice had increased α-SMA immunostaining. This change was strongly reversed in mice who received both HDM and GC021109 (Figure 5A). We used ImageJ to calculate the area of α-SMA surrounding airways and blood vessels. This demonstrated a significant increase in α-SMA area around both airways and blood vessels in HDM-exposed mice which was reversed in mice treated with HDM + GC021109 (Figure 5B).

**Figure 6:**
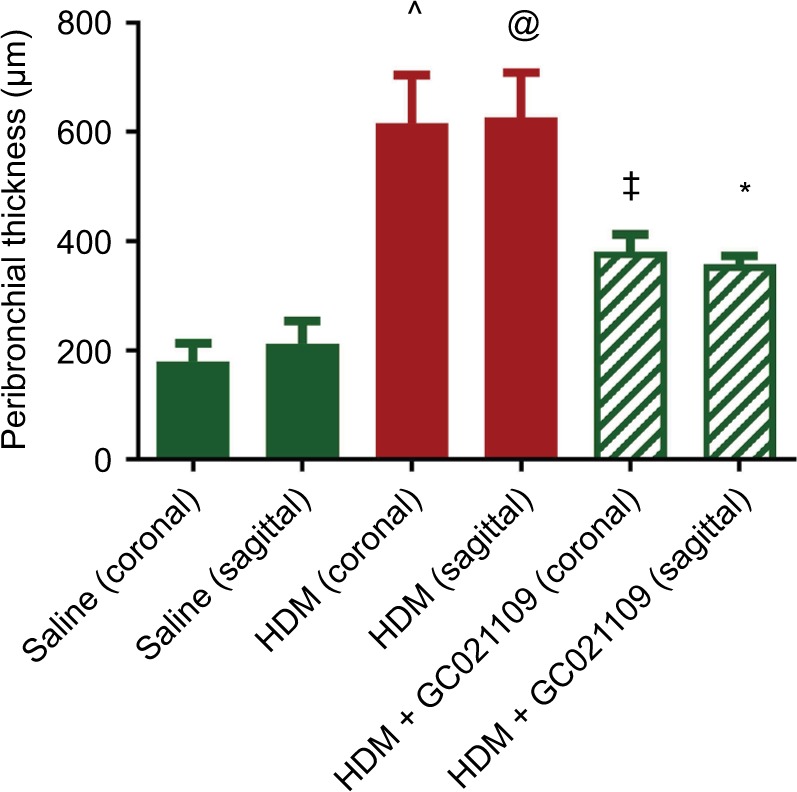
Peribronchial thickness identified by micro-CT scan is increased by HDM and ameliorated by HDM + GC021109. **Notes:** Mice treated with saline, HDM, or HDM + GC021109 (100 µg/kg weight/dose) underwent micro-CT scanning to image pulmonary airways. Images were captured at peak inspiration. Peribronchial thickness at the level of the third-generation bronchi on coronal images was measured in both the coronal and corresponding transverse axial planes. Only bronchi whose shape indicated parallel (coronal) or perpendicular (sagittal, i.e. transverse axis) orientation to the image plane were measured. Data represent mean ± SEM; N=3 mice (1–2 measurements per lung). ^@^*P*<0.10, compared to saline of same image orientation; *P<0.05, compared to HDM of same image orientation; ^^^*P*<0.005, compared to saline of same image orientation; ^‡^*P*<0.005, compared to HDM of same image orientation. **Abbreviations:** HDM, house dust mite; SEM, standard error of means; CT, computed tomography.

In addition to analyzing the α-SMA area as an index of airway remodeling, we used micro-CT scanning to measure total airway wall thickness. Third-generation bronchi were identified on coronal images. The coronal and corresponding sagittal images at that level were used for measurements of third-generation peribronchial wall thickness. The peribronchial wall thickness was increased 3-fold in HDM-exposed mice compared to controls (*P*>0.005). This increase was significantly reduced in mice given HDM + GC021109 compared to HDM alone (*P*<0.05) (Figures 6 and S3).

## Discussion

This study indicates that P2Y6R activation can play a prominent role in reducing pulmonary inflammation in the mouse HDM-induced model of asthma, in that the P2Y6R selective agonist prodrug GC021109 significantly blunted the allergen-induced inflammation and remodeling in the airways. There is disagreement regarding the role of P2Y6R in inflammation, particularly in the development of asthma. For example, studies of P2Y6R activity in vitro suggest a role in cellular response to inflammation through autocrine and paracrine actions of uracil nucleotides. Some studies indicate that extracellular nucleotides are involved in asthma pathogenesis through the activation of P2Y6R by their high affinity ligand uracil,^4^ and that upregulation of airway inflammation with allergen exposure is inhibited by blocking P2Y6R activation.^5^ Nucleotides amplify chemokine production in epithelial cells in vitro and are also present in endothelial cells and macrophages. In vitro studies indicate that UDP amplifies cytokine production by epithelium,^17^ endothelium,^18^ and macrophages.^19^ In contrast, other studies demonstrate anti-inflammatory effects in asthma of P2Y6R or its primary agonist uridine, indicating that these receptors protect the lung against allergen-induced pulmonary inflammation by inhibiting the activation of effector T-cells.^8^,^9^ A possible source of the disagreement between these various studies is a lack of sufficiently selective purinergic receptor agonists and antagonists. An alternative interpretation is that since many G protein coupled receptors internalize following either prolonged exposure to or high concentrations of their agonists, the pro-inflammatory responses are the result of receptor desensitization. Therefore, we conducted this study with a synthetic highly selective P2Y6R agonist prodrug using low doses which are known to be anti-inflammatory in neurodegenerative disorders.^20^

Airway inflammation is an important component of asthma in both its chronic status and acute exacerbations. In this allergic model of asthma, mice were sensitized with nasal HDM for 6 weeks to create a model of chronic asthma. The sensitized mice in this study showed marked evidence of airway inflammation with increased perivascular and peribronchial cellular infiltration, consistent with the findings of others who used this asthma model.^11^,^21^ We showed that a specific P2Y6R prodrug agonist significantly decreases the inflammatory response in mouse lungs, as evidenced by a decrease in the total cell count, including the eosinophil count, in LLF. Similar changes were observed in total protein concentration. In addition, histological studies showed that the P2Y6R agonist decreased the perivascular and peribronchial cellular infiltration present in the mouse HDM asthma model.

ASM cells are hyper-contractile in response to inflammatory mediators. Extracellular pyrimidines activate P2Y6R on smooth muscle cells leading to constriction.^22^ We felt it was important to determine the effect of this P2Y6R-specific agonist on smooth muscle as well as on pulmonary mechanics in our model. We found that chronic exposure of the mice to HDM increased the ASM amount. ASM cells, in addition to proliferation and hypertrophy, also synthesize and release IL-6, GM-CSF, and other mediators of inflammation in response to IL-1β and tumor necrosis factor-α.^23^ Human bronchial smooth muscle cells treated with serum from patients with atopic asthma secrete IL-5, and treatment of these cells with IL-5 provoked an asthmatic constrictor response to acetylcholine. Passive sensitization of ASM cells in culture also induced the synthesis and release of GM-CSF, IL-1β, IL-6, and IL-8. The findings in our study that treatment with GC021109 reversed the deleterious pulmonary function and the smooth muscle hyperplasia induced by HDM suggest that P2Y6R activity prevents not just the production of cytokines but also the consequences of increased cytokine production.

Allergic asthma is associated with the release of inflammatory mediators from the upper and lower airways.^24^ We found increased Th2 cytokines in LLF, consistent with previous studies using the HDM mouse model.^11^ GC021109 reduced cellular inflammation. It also reduced the levels of several Th2 cytokines that are important in asthma, exhibiting GC021109 dose–responses for IL-4, IL-5, IL-12, and G-CSF. These pro-inflammatory cytokines are commonly present in high concentrations in bronchoalveolar lavage fluid from persons with severe asthma and are associated with airway hyperresponsiveness.^1^ Additional cytokines of interest in allergen-induced asthma that were markedly increased by HDM sensitization were also markedly reduced in response to GC021109. However, these did not demonstrate a clear dose–response relationship and fell short of statistical significance. Nonetheless, these cytokine data do illustrate the strong tendency of added GC021109 to profoundly reduce the levels of specific asthma-related cytokines in HDM mice.

The innate immune response in allergy is linked to mast cells, basophils, and the innate lymphoid cell type 2 (ILC2). ILC2s respond to IL-33 and leukotrienes by producing IL-4, IL-5, IL-6, and IL-13. The specific cellular source of cytokine production in asthma is unclear, although evidence shows that allergens, including HDM, may activate airway epithelium leading to the release of mediators including IL-25, IL-33, and thymic stromal lymphopoietin, which activate ILC2. ILC2 cells in turn are a source of type 2 cytokines that contribute to eosinophilia, mucus production, polarization into alternatively activated macrophages, and B cell interactions.^25^ The ability to prevent this process by activating P2Y6Rs could be a useful tool to develop greater insight into cytokine biology in asthma.

We also found that IL-12 (p40) and G-CSF were significantly decreased in the HDM-exposed mice in response to GC021109 treatment. Like the ILC2 activating cytokines, IL-12 (p40) and G-CSF are produced by airway epithelial cells during airway inflammation. Considering the ability of P2Y6R to dampen the production of epithelial cell-derived cytokines, our study suggests that this function may be directed toward promoting chronic suppression of allergic inflammation in asthma.^26^,^27^

Purinergic receptors have been a prominent topic of investigation in asthma. P2Y6R activation may produce widely divergent responses, ranging from promotion of inflammation to downregulation of inflammation. P2Y6R is an important inhibitor of T-cell function in allergen-induced airway inflammation, based on factors such as the particular receptor ligand or the cell type involved.^28^ Mice with genetic ablation of P2Y6R exhibited an increase in the expression of pathogenic Th2 cytokines, leading to inflammatory changes in their lungs.^9^ Variable responses may also reflect the capacity of P2Y6R to heterodimerize with different receptors or a biphasic inflammatory process.^29^ The cause of inflammation may also influence the pro-inflammatory versus anti-inflammatory actions of P2Y6R activation. Cytokine production by macrophages stimulated with products of cell necrosis, but not stimulation with lipopolysaccharides, is mediated by P2Y6R activation.^30^ As allergic responses leading to inflammation in asthma are multifactorial, efforts to identify the basis of inflammatory suppression in asthma by GC021109 should be applied to multiple cell types and regulatory pathways. Additional studies of P2Y6R responses to GC021109 that examine responses in airway epithelium, smooth muscle, and resident inflammatory cells are needed to better understand how activation of the P2Y6R counteracts inflammation in asthma.

In conclusion, this study demonstrated that a prodrug specifically targeting the P2Y6R markedly improved pulmonary function dynamics, reduced ASM hyperresponsiveness, reduced airway inflammation including the presence of pro-inflammatory cytokines, and inhibited airway and vascular smooth muscle cell remodeling, all of which are major physiologic components of clinical asthma. Purinergic P2Y6R is therefore capable of exerting a positive controlling influence on airway inflammation, hyperresponsiveness, and remodeling in this mouse model of allergic chronic asthma. Additional studies that define the pathways by which these effects are exerted, and determine if similar effects are exerted on human airways are warranted. Targeting P2Y6R may prove a valuable therapeutic adjunct in asthma.

## Supplementary materials

Figure S1 LLF fluid cell differential counts noted by a hemocytometer after Wright’s staining from mice treated with saline,
**Notes:** GC021109 (10 and 100 µg/kg weight/dose), HDM, or HDM + GC021109 (10 and 100 µg/kg weight/dose). Black bars: lymphocytes; green bars: macrophages; red bars: eosinophils. Data are expressed as % total white blood cells counted, and shown as mean ± SEM; N=5 mice.
**Abbreviations:** HDM, house dust mite; SEM, standard error of means; LLF, lung lavage fluid.

Figure S2 Additional selected cytokines significantly increased in LLF fluid by HDM and normalized by the addition of GC021109.
**Notes:** These results are from the same animals and were obtained in the multiplex assay explained in the legend of Figure 4. They are shown as supplementary data as cytokines of interest in allergen-induced asthma that were markedly increased by HDM sensitization but the response to added GC021109 with HDM sensitization did not demonstrate a clear dose–response relationship. Nonetheless, these data do illustrate the tendency of added GC021109 to profoundly reduce the levels of specific cytokines in HDM mice. Specific treatment conditions are labeled along the X axis. The Y axis represents picograms/mL of IL-6, IL-17, LIF, and CXCL1, respectively. Data represent mean ± SEM; N=3–9; *P<0.05, compared to the saline controls; ^▼^*P*<0.01, compared to saline controls; ^‡^*P*<0.005, compared to saline controls; ^@^*P*<0.10, compared to HDM: ^^^*P*<0.05, compared to HDM. The HDM + GC021109 condition in the IL-6 panel had very low identical measurements; thus, no standard error or statistical test results are shown.
**Abbreviations:** HDM, house dust mite; SEM, standard error of means; LLF, lung lavage fluid; IL, interleukin; LIF, leukemia inhibitory factor; CXCL1, chemokine (C-X-C) motif ligand 1.

Figure S3 Representative micro-CT images of mice from the control, HDM, and HDM + GC021109 treatment groups.
**Notes:** Coronal (top) and transverse axis (bottom) images are shown for each condition. The horizontal line in the coronal images represents the level at which the corresponding transverse axis images were viewed and is the level of the third-generation bronchi. Arrows point to peribronchial airway thickness in third-generation airways.
**Abbreviations:** HDM, house dust mite; CT, computed tomography.
