# Supplementary figures and images for: A purinergic P2Y6 receptor agonist prodrug modulates airway inflammation, remodeling, and hyperreactivity in a mouse model of asthma
Source: J Asthma Allergy. 2018 Aug 1;11:159–71. doi: 10.2147/JAA.S151849 (PMC6078081; doi:10.2147/JAA.S151849)

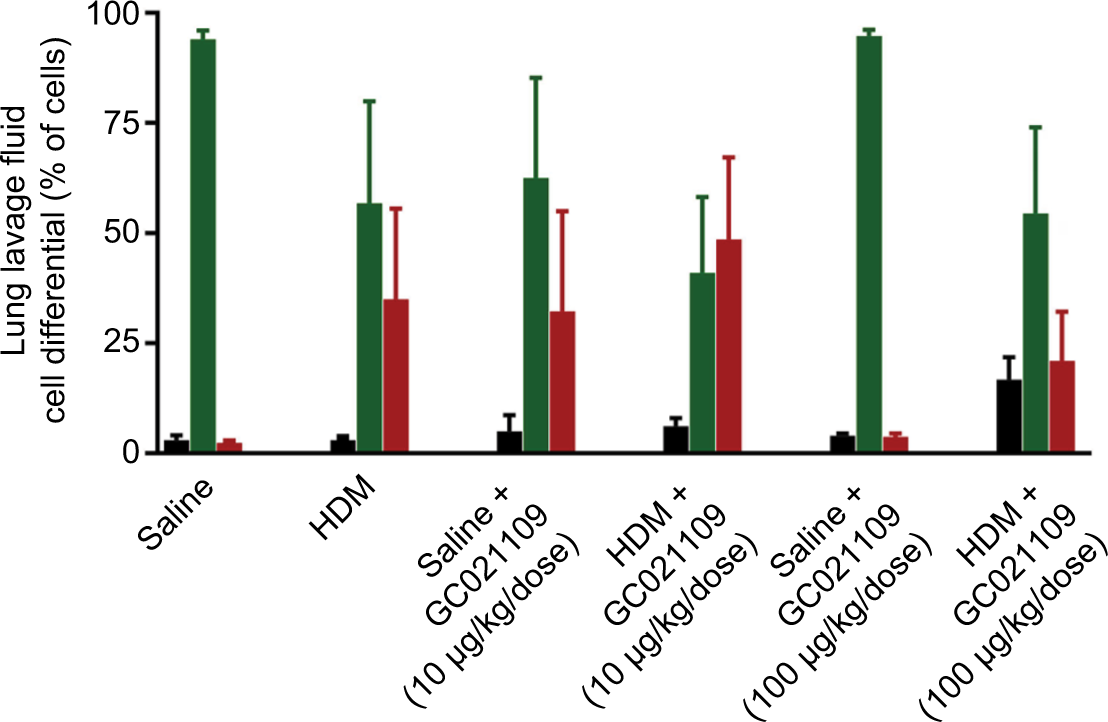

Supplement: Figure S1 — LLF fluid cell differential counts noted by a hemocytometer after Wright’s staining from mice treated with saline, Notes: GC021109 (10 and 100 µg/kg weight/dose), HDM, or HDM + GC021109 (10 and 100 µg/kg weight/dose). Black bars: lymphocytes; green bars: macrophages; red bars: eosinophils. Data are expressed as % total white blood cells counted, and shown as mean ± SEM; N=5 mice. Abbreviations: HDM, house dust mite; SEM, standard error of means; LLF, lung lavage fluid. [file jaa-11-159s1.tif]

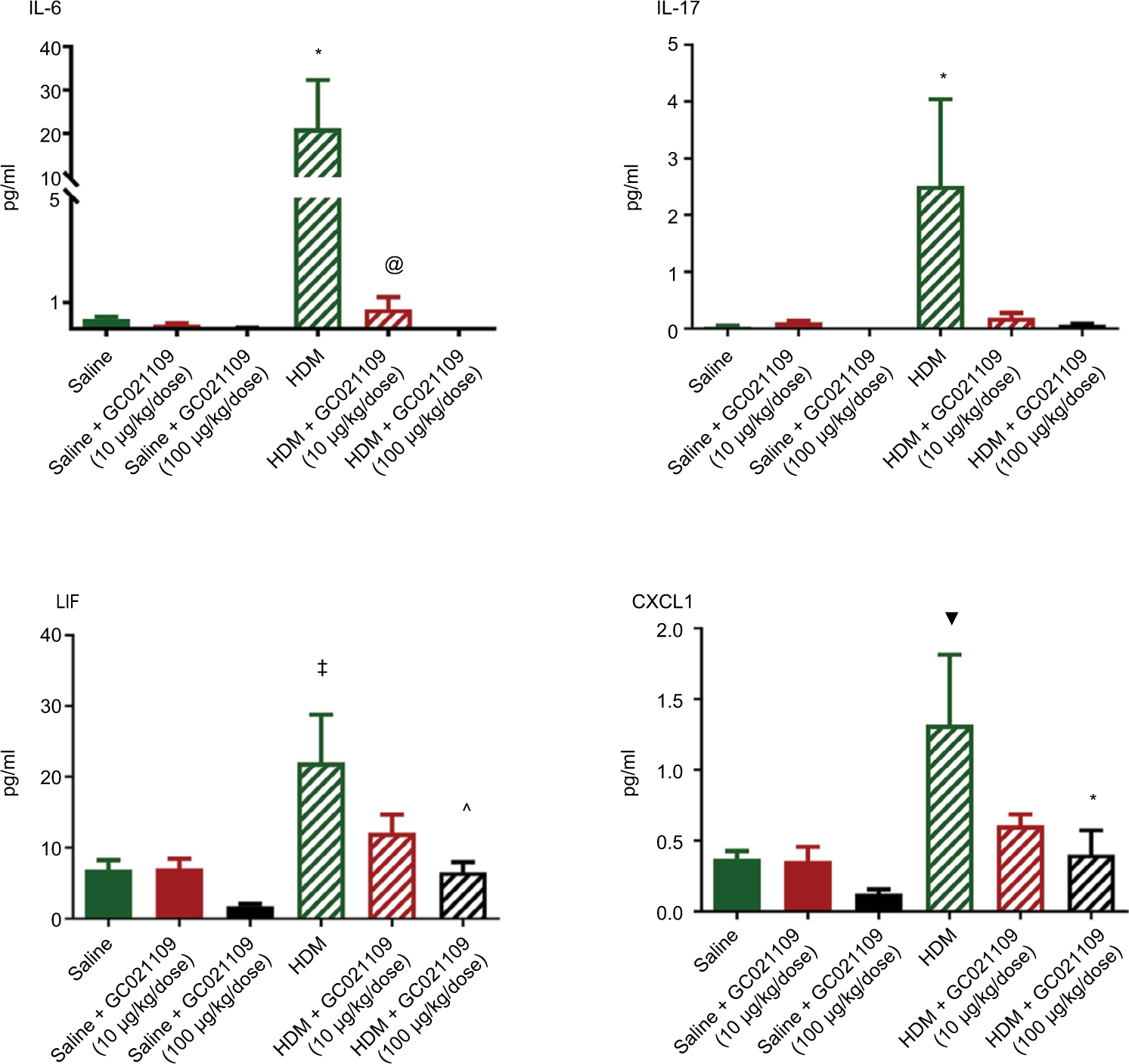

Supplement: Figure S2 — Additional selected cytokines significantly increased in LLF fluid by HDM and normalized by the addition of GC021109. Notes: These results are from the same animals and were obtained in the multiplex assay explained in the legend of Figure 4. They are shown as supplementary data as cytokines of interest in allergen-induced asthma that were markedly increased by HDM sensitization but the response to added GC021109 with HDM sensitization did not demonstrate a clear dose–response relationship. Nonetheless, these data do illustrate the tendency of added GC021109 to profoundly reduce the levels of specific cytokines in HDM mice. Specific treatment conditions are labeled along the X axis. The Y axis represents picograms/mL of IL-6, IL-17, LIF, and CXCL1, respectively. Data represent mean ± SEM; N=3–9; *P<0.05, compared to the saline controls; ▼P<0.01, compared to saline controls; ‡P<0.005, compared to saline controls; @P<0.10, compared to HDM: ^P<0.05, compared to HDM. The HDM + GC021109 condition in the IL-6 panel had very low identical measurements; thus, no standard error or statistical test results are shown. Abbreviations: HDM, house dust mite; SEM, standard error of means; LLF, lung lavage fluid; IL, interleukin; LIF, leukemia inhibitory factor; CXCL1, chemokine (C-X-C) motif ligand 1. [file jaa-11-159s2.tif]

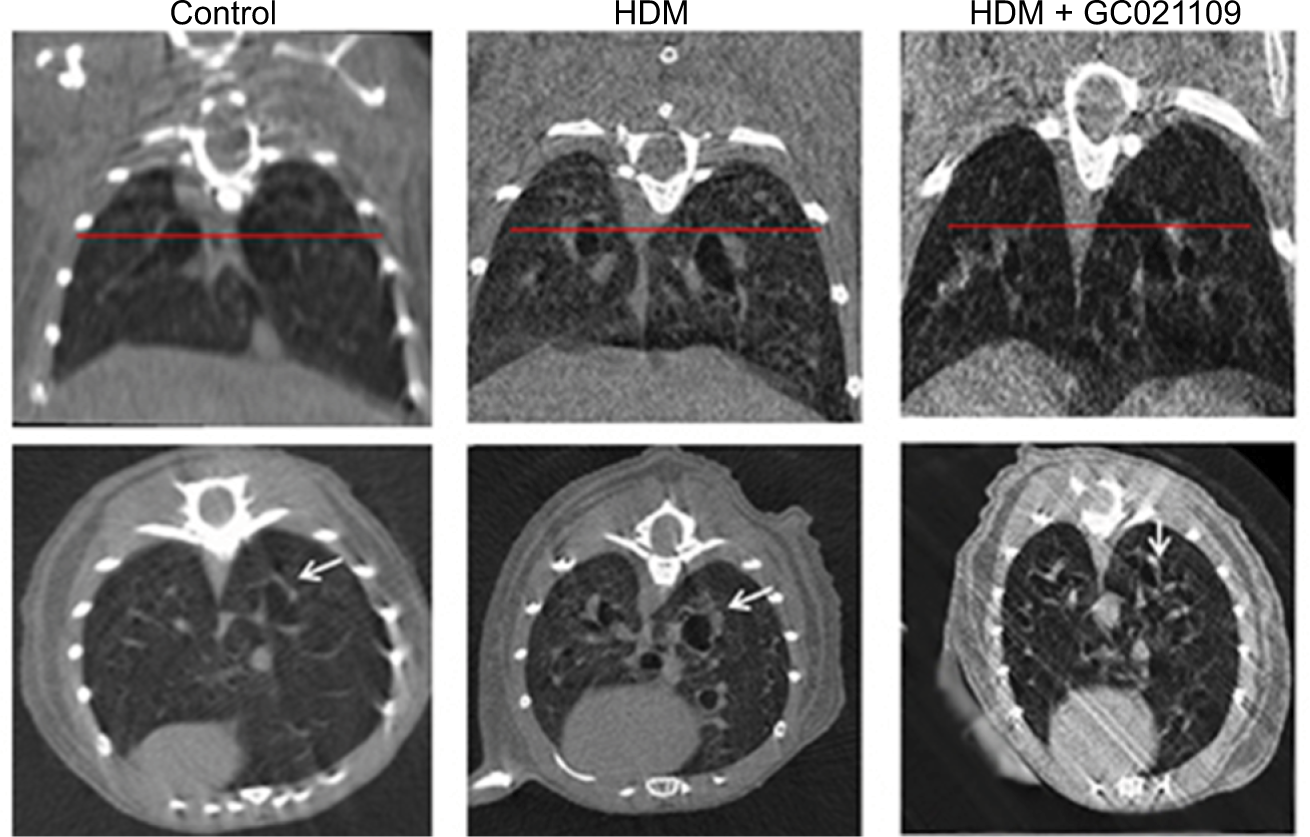

Supplement: Figure S3 — Representative micro-CT images of mice from the control, HDM, and HDM + GC021109 treatment groups. Notes: Coronal (top) and transverse axis (bottom) images are shown for each condition. The horizontal line in the coronal images represents the level at which the corresponding transverse axis images were viewed and is the level of the third-generation bronchi. Arrows point to peribronchial airway thickness in third-generation airways. Abbreviations: HDM, house dust mite; CT, computed tomography. [file jaa-11-159s3.tif]
